# Mathematical Model Describing the Influence of Geometrical Parameters of Multichannel Dies on the Limit Force of Dry Ice Extrusion Process

**DOI:** 10.3390/ma13153317

**Published:** 2020-07-25

**Authors:** Jan Górecki, Krzysztof Talaśka, Krzysztof Wałęsa, Dominik Wilczyński, Dominik Wojtkowiak

**Affiliations:** Faculty of Mechanical Engineering, Poznań University of Technology; 60965 Poznań, Poland; krzysztof.talaska@put.poznan.pl (K.T.); krzysztof.walesa@put.poznan.pl (K.W.); dominik.wilczynski@put.poznan.pl (D.W.); dominik.wojtkowiak@put.poznan.pl (D.W.)

**Keywords:** extrusion process, dry ice, multichannel die, tool design, applied mathematical model, empirical model verification

## Abstract

The article presents a formulated mathematical model that enables the determination of the required compressive force in the extrusion process of dry ice employing multichannel dies. This is the main parameter in the piston-based dry ice extrusion process. The indicated model was developed for the purpose of further improvement of the energy efficiency of this extrusion process. It allows for the determination of the value of compressive force by accounting for 12 variables related to the geometrical parameters of the die and the physical characteristics of dry ice. Furthermore, the paper also provides descriptions of the empirical study methodologies together with the results. These were carried out in order to determine the difference between the results of mathematical modeling and actual measurement results. The final part of the article presents the results of the analysis of the mathematical model’s sensitivity to the change of the physical characteristics of dry ice. The formulated tool may be employed to adapt the geometric parameters of the die in order to obtain the desired compressive force value and dry ice granulation with reduced energy consumption.

## 1. Introduction

Currently, we observe a shared interest of the international community in alleviating climate change [1,2]. Climate change is the result of, among other factors, the increase in greenhouse gas emissions, including CO_2_ [3,4]. Globally, the industry sectors involved in the manufacturing of ammonia compounds and power generation represent one of the major sources of CO_2_ emissions [5,6]. The indicated sources of greenhouse gas emissions are interpreted as highly focused sources, where utilization of installations for capturing CO_2_ is justified both by social and economic reasons [5,7]. Very often, the recovered gas becomes a side product of the process and is subject to further processing by the source entity [8]. However, the amount of recovered material usually exceeds the on-site capacity for its utilization, and therefore it is handed over to interested recipients for utilization, e.g., in the enrichment process of recovered oil or in urea system installations [5,9].

On the market, it has been observed that there are recipients interested in liquid carbon dioxide for its use in crystallization [10,11]. The material in this state is characterized by a temperature of −78.5 °C and sublimation in normal conditions [12,13]. Due to the indicated peculiar characteristics, it is often referred to by its common name “dry ice” [14,15,16]. It is used in material transportation [17,18], surface cleaning [19,20,21,22,23,24], and disinfection [16,25], among other uses. However, in the process of crystallization of liquid CO_2_, a fragmented material is obtained [26], which results in a short sublimation time and low efficiency of its use in, e.g., refrigeration processes [27]. Therefore, in order to extend the sublimation time of the material as well as to improve its use efficiency, it is compacted and delivered, e.g., in the form of pellets [28].

**Figure 1:**
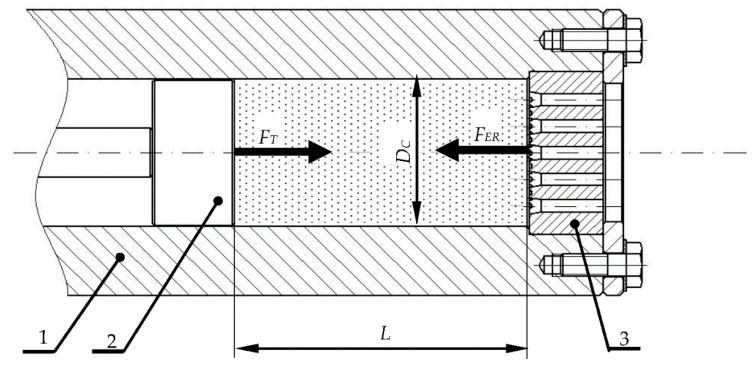
Extrusion system assembly utilizing the crank-piston technique: 1—working chamber, 2—compacting piston, 3—multichannel die, *L*—agglomerated deposit height, *D_C_*—working chamber diameter, *F_ER_*—extrusion resistance force of die, *F_T_*—force applied by the piston [10].

Pelletized dry ice is obtained through extrusion by machines employing the piston-based working system, as shown in Figure 1. As a result of the expansion of liquid CO_2_ in a closed, cylindrical working chamber (1), the material crystallizes. Subsequently, the motion of the compacting piston (2) compresses the fragmented dry ice. The process continues until the force applied by the piston *F_T_* is balanced by the resistance force *F_ER_* necessary to be overcome for the material to be pressed through the die channels. The resistance force value *F_ER_* affects the density of the product, where in order to improve the utilization efficiency of the compressed carbon dioxide, it is justified to achieve the highest possible value of the indicated material parameter [27]. The available subject literature provides formulated algebraic models describing the relation between the limit compaction force and the geometrical parameters of the convergent die channel [29,30].

The models available in the subject literature do not relate to the extrusion of fragmented material with the utilization of multichannel dies, which are successfully employed for the extrusion of carbon dioxide. In order to fill in the gaps in the subject literature, work has been undertaken to formulate an algebraic model that makes it possible to establish the relations between the geometrical parameters of the multichannel die and the resistance force value.

## 2. Theoretical Analysis of the Extrusion Process Utilizing Dies

The analysis was carried out based on the model available in the subject literature [29]. It assumes that the compaction force necessary to carry out the extrusion process in the single convergent circular symmetrical channel results from the energy dissipation balance at the forming section *P_D_*, which is necessary to overcome the frictional resistance *P_µ_* as well as the value of linear velocity of the material at the beginning of the duct. This relationship is expressed with the following formula:

(1) $$F_{ER}\times v_{in}=P_{D}+P_{µS}+P_{µC}$$

Based on Huber’s hypothesis [29], the substitute yield strength was determined as $\sqrt{3}$*τ_a_*. Hence, the dissipated power value *P_D_*, as a function of the geometric and kinematic parameters of the process in an axial-symmetrical channel can be described with the following Equation:

(2) $$P_{D}=\sqrt{15}\times \tau_{a}\times v_{in}\times \frac{D_{in}^{2}}{4}\times ln\frac{D_{in}}{D_{out}}$$

Based on the product displacement value, an algebraic formula was established to describe the variance in energy dissipation due to friction in a symmetrically convergent forming channel *P_μS_*:

(3) $$P_{µs}=\int_{S_{s}}^{}{µ}_{T}\times \tau_{a}\times w_{k\;}dS_{s}$$

where *S_S_* is the surface area of the side of the convergent section. Its value can be described with the following formula:

(4) $$S_{s}=\int_{S_{s}}^{}2\pi \times R\left(z\right)dS_{s}$$

After integration and transformation of the above equations (refer to Equations (3) and (4)) we arrive at the following equation:

(5) $$P_{µS}=\mu_{T}\times \tau_{a}\times \frac{v_{in}\times D_{in}^{2}}{cos\alpha \times D_{out}^{2}}\times \frac{2\pi }{cos\alpha }\left(\frac{D_{in}}{2}\times b-\frac{b^{2}}{2}\times tan\alpha \right)$$

Subsequently, frictional power in the cylindrical section of the forming channel was determined similarly, using the following Equation:

(6) $$P_{\mu C}=\int_{S_{C}}^{}\mu_{T}\times \tau_{a}\times v_{in}\;dS_{C}$$

where *S_C_* is the surface area of the side of the cylindrical section, which can be described with the following Equation:

(7) $$S_{C}=\int_{\theta =0}^{2\pi }\int_{z=0}^{a}\frac{D_{out}}{2}dzd\theta =\int_{\theta =0}^{2\pi }\int_{z=0}^{a}\frac{D_{in}}{2}-b\times tan\alpha \;dzd\theta .$$

After integration and transformation of Equations (6) and (7), we arrive at the following equation:

(8) $$P_{\mu C}=\mu_{T}\times \tau_{a}\times v_{in}\times \frac{D_{out}^{2}}{4\times D_{in}}\times a$$

After making transformations to the model (Equation (1)), we arrive at the relation binding the indicated force value to the geometrical parameters of the single channel die, as well as the physical characteristics of the compacted material (such as the shear stress of the pellet *τ_a_* and the static friction coefficient *μ_T_*). The relationship is expressed with the following formula:

(9) $$F_{ER}=\tau_{a}\frac{D_{in}{}^{2}}{4}\left(\sqrt{15}\times ln\frac{D_{in}}{D_{out}}+2\pi \times \mu_{T}\left(\frac{4}{cos^{2}\alpha \times D_{out}{}^{2}}\left(\frac{D_{in}}{2}\times b-\frac{b^{2}}{2}tan\alpha \right)+\frac{2\alpha }{D_{out}}\right)\right)$$

**Figure 2:**
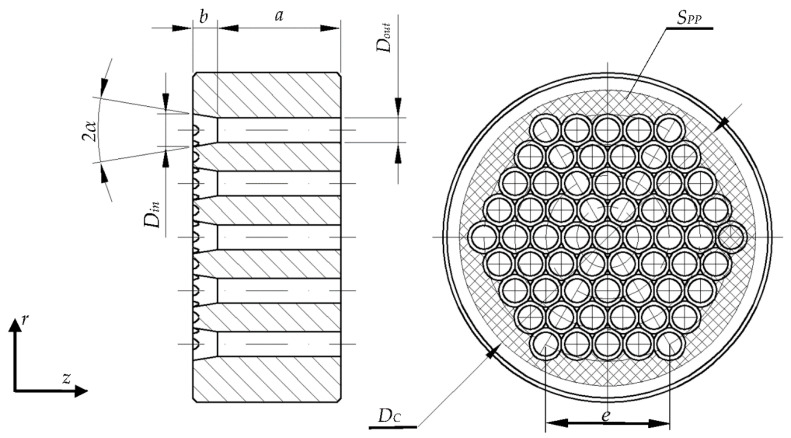
EMultiorifice die: α—convergence angle of the conical section, *a*—length of the conical section, *b*—length of the cylindrical section, *D_in_*—input diameter of the conical section, *D_out_*—output diameter of the cylindrical section, *e*—side length of the hexagon on which the channels are distributed, *S_PP_*—surfaces perpendicular to the direction of the piston displacement vector, *D_C_*—a diameter of the compaction chamber.

The theoretical analysis of the extrusion process utilizing dies with n symmetrical circular forming channels, as shown in Figure 2, calls for developing the model provided in the subject literature to include the following constituents of the balance: energy required to separate the extruded material *P_T_* [27,31], energy dissipated during deformation of the extruded material *P_DPP_* on the surface perpendicular to axis *z S_PP_* [10], and energy dissipated as a result of friction by the material as the extruded material moves inside the working chamber *P_µC_*. The developed relationship can be expressed as:

(10) $$F_{ER}\times v_{in}=n\times \left(P_{D}+P_{\mu }\right)+P_{T}+P_{DPP}+P_{\mu C}$$

Analogous to the earlier model, after transforming the formula, we arrive at the relationship binding the value *F_ER_* to the geometrical parameters of the channel (where additionally the following variables were accounted for: the number of channels *n*, length and diameter of the working chamber *D_C_*, side length of the hexagon on which the orifices *e* were distributed, and number of channels arranged on a hexagon *n_G_*), as well as the material and the process, i.e., the length of the extruded material before it is introduced into the die channels *l_T_*. The resulting relationship is expressed as

(11) $$\begin{array}{c} F_{ER}=\tau_{a}(n\;\frac{D_{in}{}^{2}}{4}\left(\sqrt{15}\times ln\frac{D_{in}}{D_{out}}+2\pi \;\mu_{T}\left(\frac{4}{cos^{2}\alpha \;D_{out}{}^{2}}\left(\frac{D_{in}}{2}b-\frac{b^{2}}{2}tan\alpha \right)+\frac{2\alpha }{D_{out}}\right)\right)\\ +l_{T}\;n\;D_{in}+\mu_{T}\left(\pi \frac{D_{C}{}^{2}}{4}-\frac{3e^{2}\sqrt{3}}{2}+\frac{\pi \;n_{G}\;D_{in}{}^{2}}{8}\right)+\pi \;\mu_{T}\;D_{C}\;l_{T}) \end{array}$$

## 3. Empirical Verification of the Developed Model

The formulated model was verified by carrying out the program of empirical data. The study was based on the methodology provided in the subject literature [32].

Experiments carried out for the purpose of the study used four dies with parameters as provided in Table 1.

Analogous to the methodology provided in the subject literature, the study employed the MTS Insight testing machine (MTS System Corporation, Eden Prairie, MN, USA) equipped with a 50 kN tensometric sensor. During the examination, the force value provided at the compressing piston *F_P_* and crossbeam displacement *x* was measured and registered with a constant frequency of 10 Hz. The study was performed at a constant velocity value equal to 9 mm/s. The measurements were repeated 10 times for every one of four dies described above.

**Figure 3:**
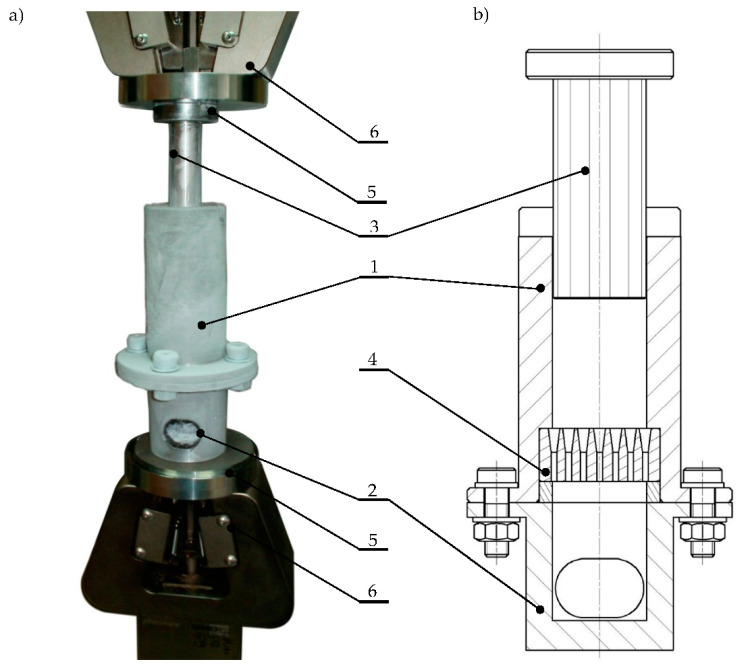
Measuring unit: (**a**) MTS machine grips with measuring head and alignment system; (**b**) cross-section of the measuring head: 1—compaction chamber, 2—head base, 3—piston, 4—multichannel forming die, 5—alignment jig, 6—jaws of testing machine [27].

The study employed the testing station that was designed and built as shown in Figure 3.

During testing, the compaction chamber (1) was filled with fragmented dry ice. The assembled unit was placed between the testing machine grips (7). After taring the value of the measurement signal, the movement of the piston (3) was forced, which was accompanied by compaction of fragmented dry ice until the values of available force at the piston *F_P_* and resistance force *F_ER_* were equal. Then, the extruded material was moved further through the forming die channels (4).

The results of the examination were used to determine the maximum force value available at the piston *F_T_* and its corresponding compacting piston displacement value for each of the four tested dies.

The point at which the force applied at the piston *F_T_* is at its maximum value is related with overcoming the resistances associated with the forming of the compacted material in the die. This method of determination of the maximum value of force at piston *F_T_* is tantamount to determining the empirical value of the resistance force *F_ER_* at the same point in the examined die. The information on the position of this point was furthermore used to determine the value of the length of the cutting edge along the axis of the compaction chamber *l_T_*, which was utilized further in this paper for calculating the value of resistance force *F_ER_* with utilization of the analytical model.

As an estimator of the sought limit axial force value, the average value *F_ER_^avr^* was assumed. For every value, measurement inaccuracy was determined, which was equal to the standard deviation of the results. The estimator values *F_ER_^avr^* and *l_T_* are provided in Table 2.

Based on the geometrical properties provided in Table 1 above, the extrusion resistance force *F_ER_^A^* was calculated for the examined dies. The results of the analysis are provided in Table 3. At the determination of the value of the relative error of the model *δ*, standard deviation *σ* was included in the consideration in relation to the value *F_ER_^avr^*. The final relationship was expressed with the following Equation:

(12) $$\delta =\frac{F_{ER}{}^{avr}\pm \sigma -F_{ER}{}^{A}}{F_{ER}{}^{avr}\pm \sigma }\times 100\%$$

The determined error values together with the results of the analysis and examination are presented in the table below.

The results obtained from the calculations for the MCD-0, MCD-2, and MCD-3 dies are within the dispersion range of the study results.

Based on the results of the model error analysis and the performed examination, it was determined that the established mathematical model makes it possible to derive the approximate force value *F_ER_*. Therefore, the results obtained from the analytically formulated model may be used for the purpose of designing machines used for the compaction of crystallized carbon dioxide with the utilization of multichannel dies.

## 4. Susceptibility of the Algebraic Model to Variance in Physical Characteristics of the Extruded Dry Ice

**Figure 4:**
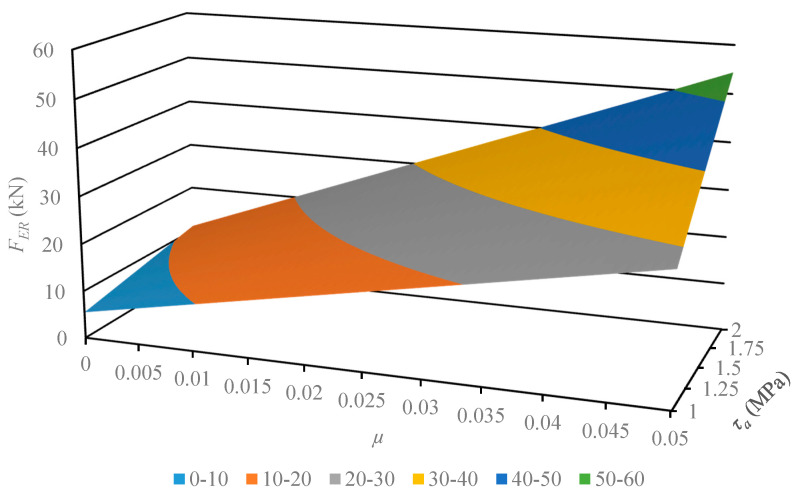
Characteristics of the limit value extrusion force *F_ER_* of dry ice in a function of material parameters (shear strength *τ_a_* and friction coefficient *μ*), in the case of using the MCD-0 multichannel die.

**Figure 5:**
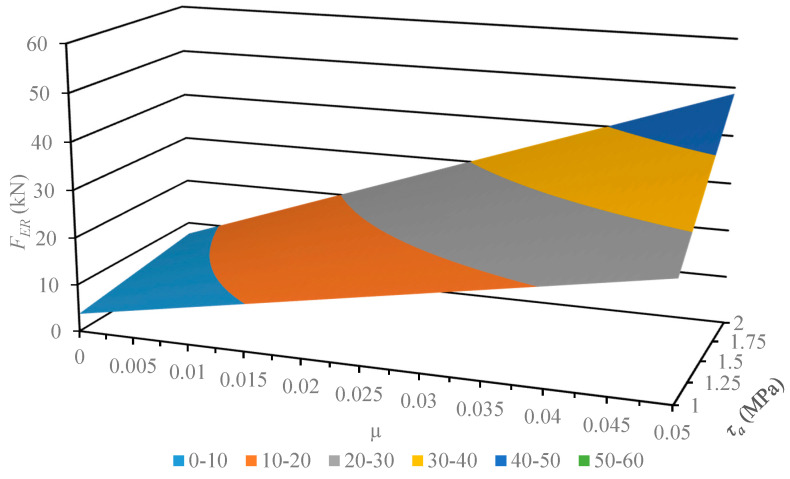
Characteristics of the limit value extrusion force *F_ER_* of dry ice in a function of material parameters (shear strength *τ_a_* and friction coefficient *μ*), in the case of using the MCD-1 multichannel die.

**Figure 6:**
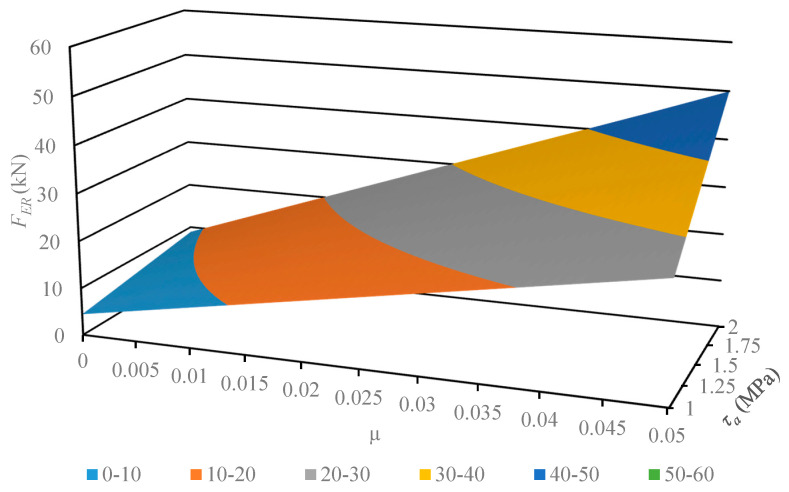
Characteristics of the limit value extrusion force *F_ER_* of dry ice in a function of material parameters (shear strength *τ_a_* and friction coefficient *μ*), in the case of using the MCD-2 multichannel die.

**Figure 7:**
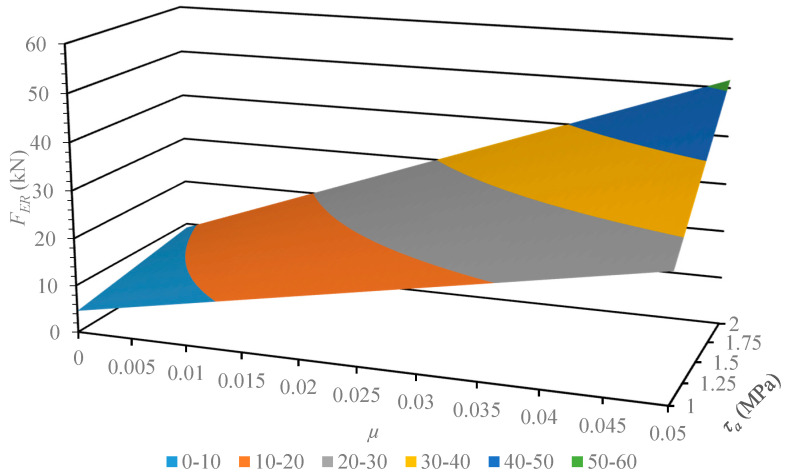
Characteristics of the limit value extrusion force *F_ER_* of dry ice in a function of material parameters (shear strength *τ_a_* and friction coefficient *μ*), in the case of using the MCD-3 multichannel die.

This section describes the next step of this work, where the developed and verified algebraic model was used for the analysis of its susceptibility to variance in the physical parameters of the material. The geometrical parameters of the model describing the multichannel dies employed earlier remained unchanged. The values subject to change were pellet shear strength *τ_a_*, in the range 1–2 MPa, and frictional coefficient *μ*, in the range 0.001–0.01. The results of the computation are displayed in Figure 4, Figure 5, Figure 6 and Figure 7.

The developed characteristics demonstrate a linear influence of both parameters on the *F_ER_* value, which is substantiated by the calculated correlation coefficient value equal to 1. In order to compare the model susceptibility to variance in both parameters under analysis, the model gradient value was determined in the examined range. The results are provided in Table 4 and Table 5.

The results of the model susceptibility analysis provided in the tables indicate that it is approximately 100 times more susceptible to variance in *μ* value than *τ_a_* value. It should be noted that is it easier to modify the value of the friction coefficient, which is related to the roughness of channel surfaces. The value of shear stress is related to the density of compacted material, where it is known as a quality factor of the product. Therefore, researchers in the future should focus on the influence of channel surface roughness and geometrical parameters on the value of extrusion force *F_ER_*. This will be necessary to reduce the value of limit extrusion stress to an effective level, which equals 14 MPa [27].

## 5. Summary

The formulated algebraic model was verified empirically. This makes it possible to estimate the value of limit force necessary to perform the extrusion process of dry ice utilizing multichannel dies with a known error.

Models that are known in the literature allow calculation of the extrusion force when single-channel dies are used in the machine. The authors of this paper could not find models that allow calculation of the extrusion limit force in cases when multichannel dies are used. Where multichannel dies are a standard solution in the process of extrusion, dry ice pellets are 3 mm in diameter or less.

The preliminary analysis of model susceptibility to the variability of physical parameter values of compacted crystallized carbon dioxide indicates that the force *F_ER_* value is more materially influenced by the value of the frictional coefficient *μ* than its shear strength *τ_a_*. This justifies the significant difference between the calculated and actual values. The authors did not measure the porosity of the sides of forming channels in the dies used in the experiment during empirical testing. However, this does not reduce the utility of the developed model.

The formulated model could be used to calculate the extrusion limit force of the designed die. It could be also used in optimization research whose main goal will be reducing the value of the limit extrusion stress to the effective value of 14 MPa [27]. In the available literature focused on the optimization of the tool shape, the optimization algorithms are well described [33,34,35]. It is likely that in the next stage of research they will be implemented in studies concerning the optimization of the die channel shape.

The formulated model may be developed further to enable the determination of the value of limit strength of the process utilizing multichannel dies with a shape other than conical or cylindrical.

## Figures and Tables

**Table 1:** Geometrical parameters of the dies.

Name of Die	*n*	*D_out_* (mm)	*D_in_* (mm)	*α* (ᵒ)	*a* (mm)	*b* (mm)	*D_C_* (mm)	*e* (mm)	*n_G_*
**MCD-0**	61	3	4.06	10	15	3	36	15	24
**MCD-1**	37	3	5.12		12	6		15	18
**MCD-2**	37	4.5	5.56		15	3		15	18
**MCD-3**	37	4.5	5.56		22	3		15	18

**Table 2:** Estimated values of limit axial force.

Name of the Die	MCD-0	MCD-1	MCD-2	MCD-3
*F_ER_^avr^* (kN)	23.1 ± 2.3	16.6 ± 2.4	18.45 ± 0.69	19.67 ± 0.82
*l_T_* (mm)	21.0	17	20.5	21

**Table 3:** Analysis results for *F_ER_* force value of forming dies.

Name of the Die	MCD-0	MCD-1	MCD-2	MCD-3
*F_ER_^A^* (kN)	22.12	18.70	19.76	20.38
*F_ER_^avr^* (kN)	23.1 ± 2.3	16.6 ± 2.4	18.45 ± 0.69	19.67 ± 0.82
*δ* (%)	−6.3–12.9	−31.69–1.58	−11.26–3.24	−8.12–0.54

**Table 4:** The characteristic gradient value describing the variance in limit force as a function of *τ_a_*, for different values of *μ.*

Gradient of Function	*μ* = 0.001	*μ* = 0.002	*μ* = 0.005	*μ* = 0.0075	*μ* = 0.01
$\nabla f^{MCD-0}\left(\tau_{a}\right)=\frac{d\;f^{MCD-0}}{d\;\tau_{a}}$ (kN)	9.8	8.8	7.7	6.4	5.9
$\nabla f^{MCD-1}\left(\tau_{a}\right)=\frac{d\;f^{MCD-1}}{d\;\tau_{a}}$ (kN)	7	6.8	5.8	4.5	4.1
$\nabla f^{MCD-2}\left(\tau_{a}\right)=\frac{d\;f^{MCD-2}}{d\;\tau_{a}}$ (kN)	8.6	7.5	6.5	5.2	4.9
$\nabla f^{MCD-3}\left(\tau_{a}\right)=\frac{d\;f^{MCD-3}}{d\;\tau_{a}}$ (kN)	8.8	7.7	6.7	5.4	5.0

**Table 5:** The characteristic gradient value describing the variance in limit force as a function of *μ*, for different values of *τ_a_*.

Gradient of Function	*τ_a_* = 1 (MPa)	*τ_a_* = 1.1 (MPa)	*τ_a_* = 1.5 (MPa)	*τ_a_* = 1.75 (MPa)	*τ_a_* = 2 (MPa)
$\nabla f^{MCD-0}\left(\mu \right)=\frac{d\;f^{MCD-0}}{d\mu }$ (kN)	868.8	760.2	651.6	477.8	434.4
$\nabla f^{MCD-1}\left(\mu \right)=\frac{d\;f^{MCD-1}}{d\mu }$ (kN)	826.8	723.5	620.1	454.8	413.4
$\nabla f^{MCD-2}\left(\mu \right)=\frac{d\;f^{MCD-2}}{d\mu }$ (kN)	821.4	718.6	616.1	451.8	410.7
$\nabla f^{MCD-3}\left(\mu \right)=\frac{d\;f^{MCD-3}}{d\mu }$ (kN)	851.5	745.0	638.6	768.3	425.7
